# Supplementary figures and images for: Correlated Responses to Selection for Intramuscular Fat on the Gut Microbiome in Rabbits
Source: Animals (Basel). 2024 Jul 16;14(14):2078. doi: 10.3390/ani14142078 (PMC11273372; doi:10.3390/ani14142078)

**a**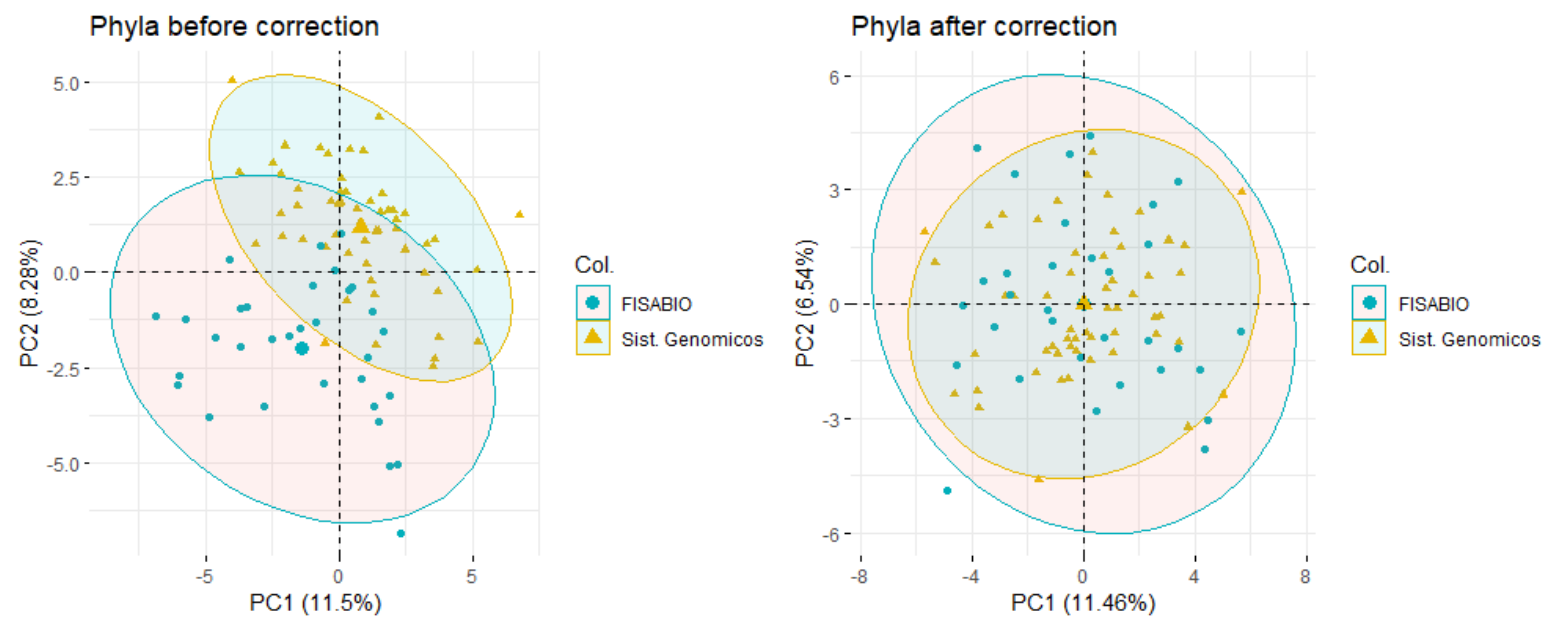**b**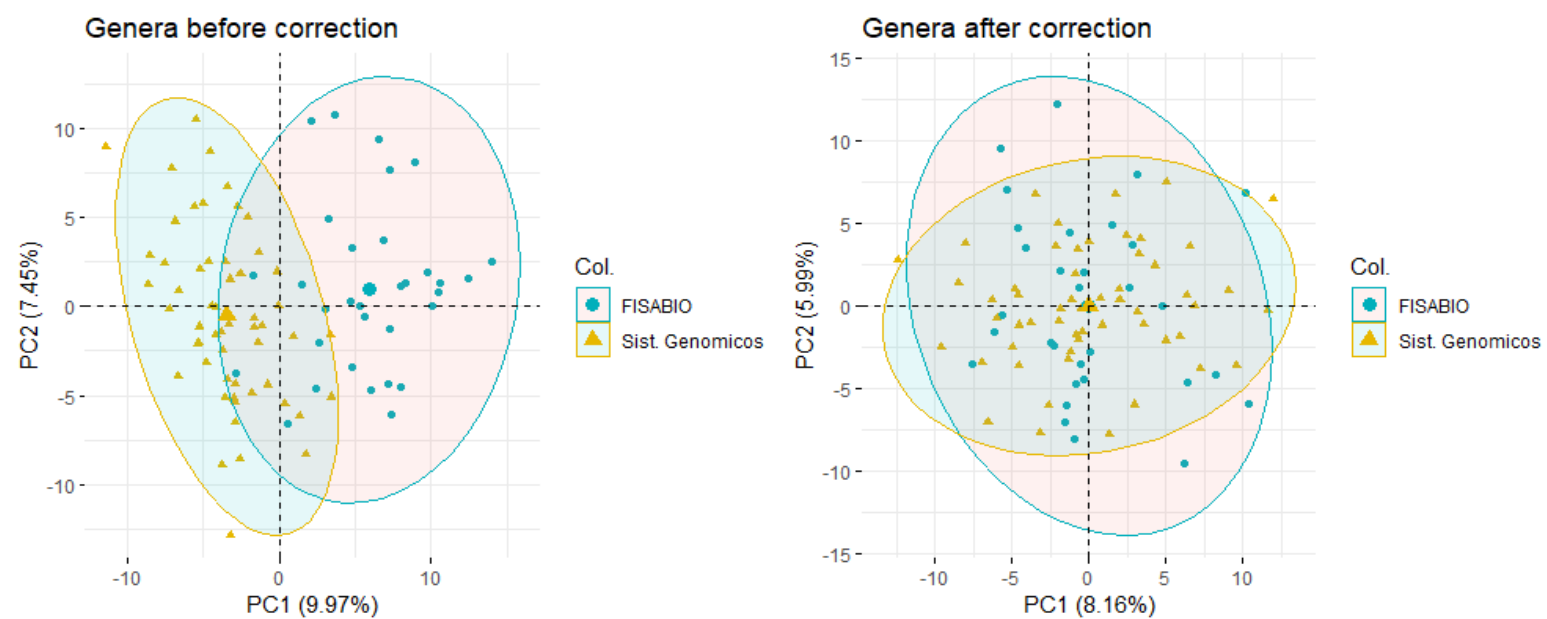

Supplement: Supplementary file 1 [file animals-14-02078-s001.zip › Figure S1.pdf]

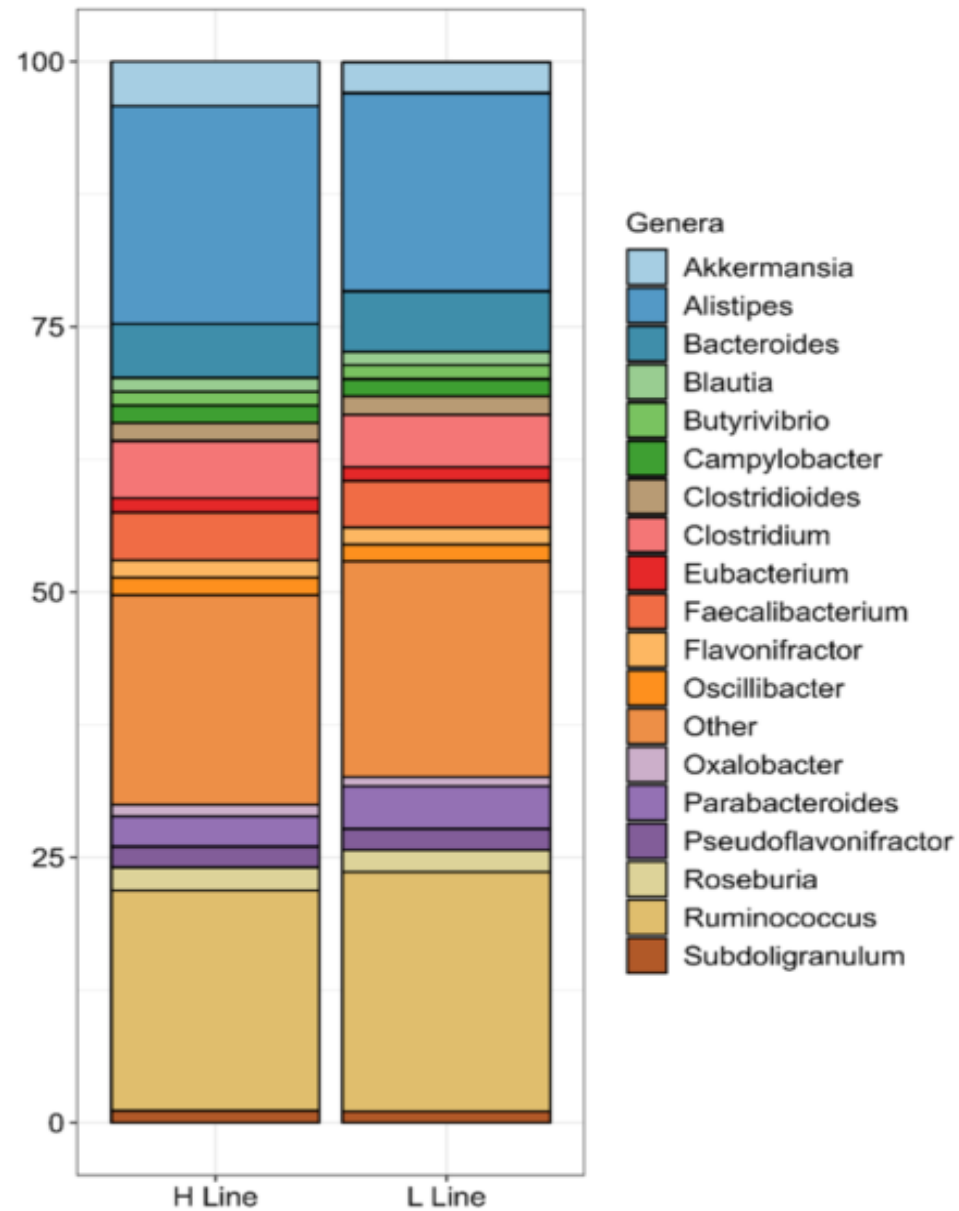

Supplement: Supplementary file 1 [file animals-14-02078-s001.zip › Figure S2.pdf]

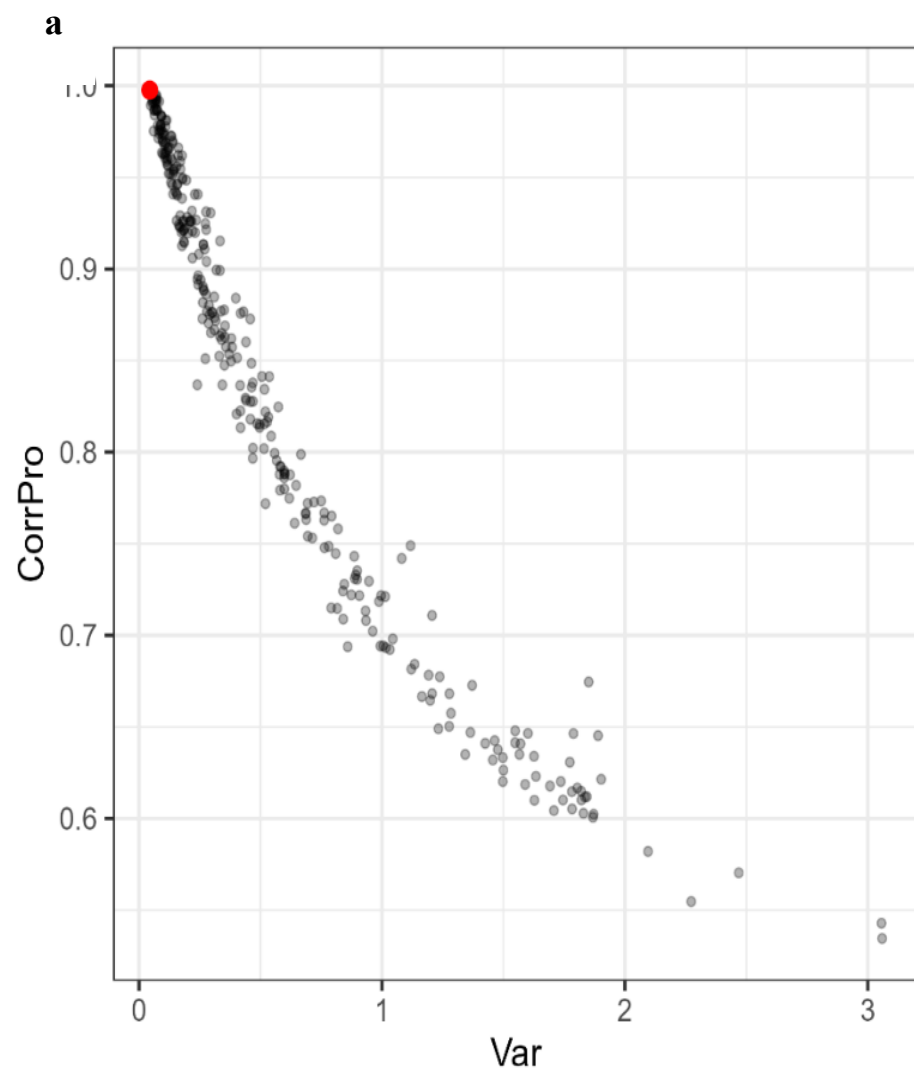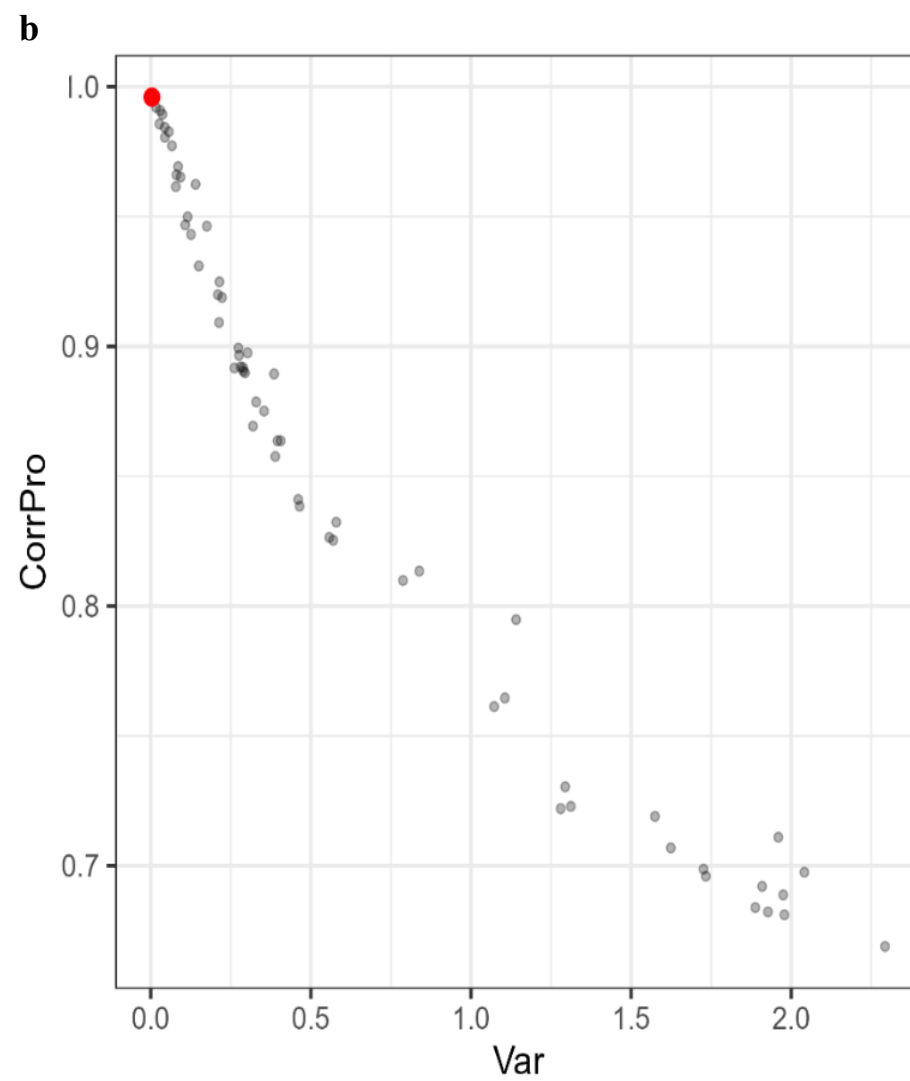

Supplement: Supplementary file 1 [file animals-14-02078-s001.zip › Figure S3.pdf]

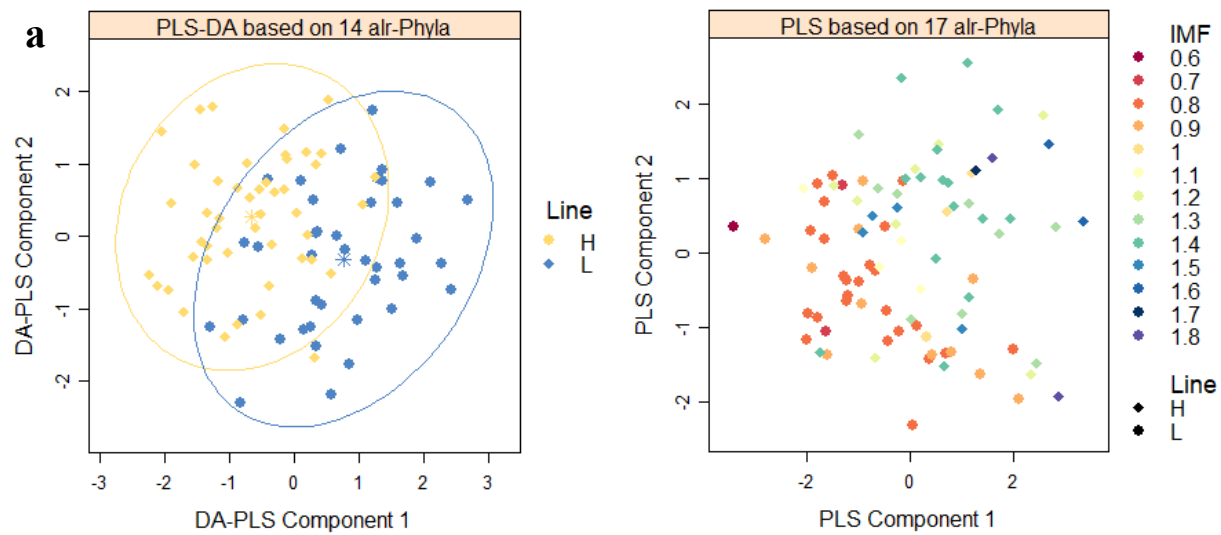

**b** PLS (17) PLS-DA (14)

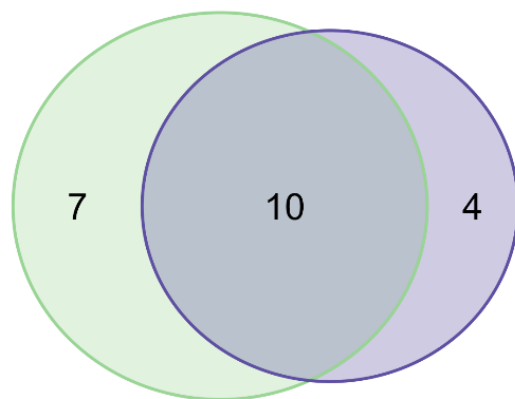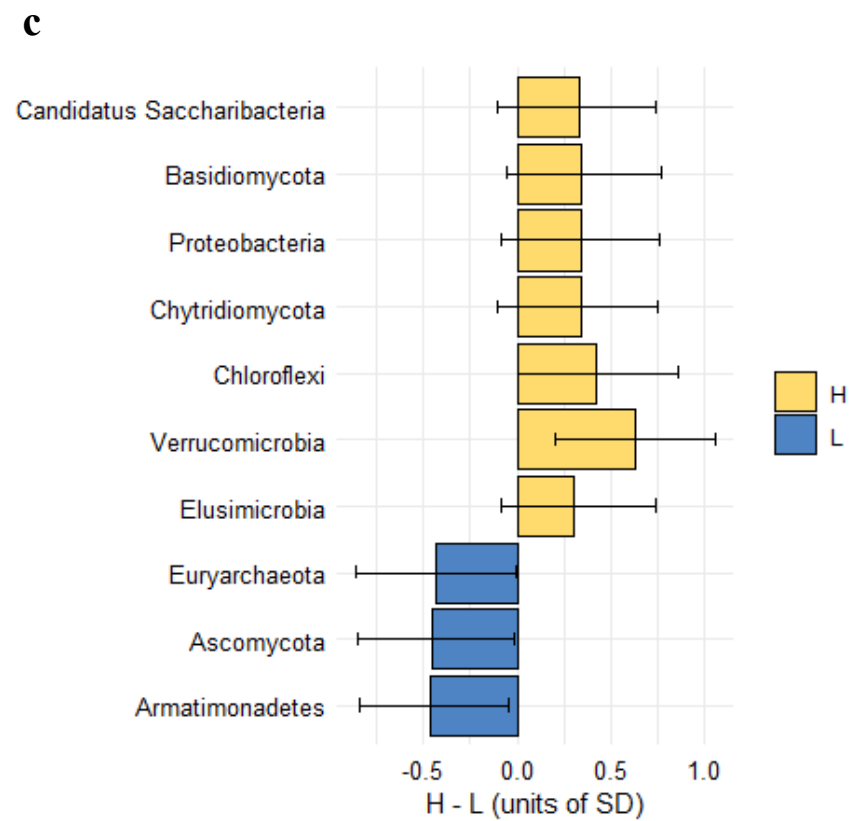

Supplement: Supplementary file 1 [file animals-14-02078-s001.zip › Figure S4.pdf]
